# Preoperative Erythrocyte Sedimentation Rate Independently Predicts Overall Survival in Localized Renal Cell Carcinoma following Radical Nephrectomy

**DOI:** 10.1155/2012/524981

**Published:** 2012-07-29

**Authors:** Brian W. Cross, Timothy V. Johnson, Austin B. DeRosa, Kenneth Ogan, John G. Pattaras, Peter T. Nieh, Omer Kucuk, Wayne B. Harris, Viraj A. Master

**Affiliations:** ^1^Department of Urology, Emory University, Atlanta, GA 30322, USA; ^2^Department of Medical Oncology, Emory University, Atlanta, GA 30322, USA; ^3^Emory Winship Cancer Institute, Emory University, Atlanta, GA 30322, USA

## Abstract

*Objectives*. To determine the relationship between preoperative erythrocyte sedimentation rate (ESR) and overall survival in localized renal cell carcinoma (RCC) following nephrectomy. *Methods*. 167 patients undergoing nephrectomy for localized RCC had ESR levels measured preoperatively. Receiver Operating Characteristics curves were used to determine Area Under the Curve and relative sensitivity and specificity of preoperative ESR in predicting overall survival. Cut-offs for low (0.0–20.0 mm/hr), intermediate (20.1–50.0 mm/hr), and high risk (>50.0 mm/hr) groups were created. Kaplan-Meier analysis was conducted to assess the univariate impact of these ESR-based groups on overall survival. Univariate and multivariate Cox regression analysis was conducted to assess the potential of these groups to predict overall survival, adjusting for other patient and tumor characteristics. *Results*. Overall, 55.2% were low risk, while 27.0% and 17.8% were intermediate and high risk, respectively. Median (95% CI) survival was 44.1 (42.6–45.5) months, 35.5 (32.3–38.8) months, and 32.1 (25.5–38.6) months, respectively. After controlling for other patient and tumor characteristics, intermediate and high risk groups experienced a 4.5-fold (HR: 4.509, 95% CI: 0.735–27.649) and 18.5-fold (HR: 18.531, 95% CI: 2.117–162.228) increased risk of overall mortality, respectively. *Conclusion*. Preoperative ESR values represent a robust predictor of overall survival following nephrectomy in localized RCC.

## 1. Introduction

Over 50,000 Americans are diagnosed with renal cell carcinoma (RCC) each year, approximately 30% of whom will ultimately develop metastatic progression of their disease despite apparent curative nephrectomy for localized cancer at the time of clinical presentation [1, 2]. Metastatic RCC, untreated, has a dismal 5-year survival rate of <10% and a median overall survival of less than one year [3–6]. As such, there has been a long-standing interest in accurately identifying those patients most likely to suffer from postoperative disease progression, and much research in recent years has focused on the development of prognostic models to aid in surveillance strategies and patient counseling. Currently, the most commonly used tool to predict outcome in RCC is the TNM staging system. However, there is considerable overlap in survival between stages [5], and this has fostered the search for other prognostic markers to more clearly stratify those patients in whom a poor outcome can be expected. 

 Recently, efforts at identifying markers of disease progression in RCC have focused on the readily available and cost-effective clinical indices of preoperative laboratory values [7]. It is becoming increasingly clear that neoplastic progression depends on an orchestrated interface between tumor biology and the host inflammatory response [8]. The systemic inflammatory response, as represented by aberrations in circulating levels of acute-phase reactants, has previously been shown to be a predictor of poor overall survival in a variety of advanced malignancies [9–11]. Indeed, we and multiple other groups have recently shown preoperative C-reactive protein (CRP) levels are an independent predictor of metastasis and mortality following extirpative surgery for localized RCC [12].

 The determination of the erythrocyte sedimentation rate (ESR) is by a simple and inexpensive laboratory test introduced by Westergren in 1921. It measures the distance erythrocytes have fallen after one hour in a vertical column of anticoagulated blood under the influence of gravity [13]. Though its clinical usefulness as a diagnostic tool has diminished as more intricate methods of analysis have emerged, it remains paramount in the specific diagnosis of a few conditions, including temporal arteritis, polymyalgia rheumatica, and rheumatoid arthritis. Moreover, an extreme elevation is mostly associated with infection or malignancy [13]. Numerous studies over three decades have substantiated the prognostic utility of ESR in patients with RCC [7, 14–23], with recent data from the Mayo Clinic showing elevated ESR levels predicting the presence of aggressive disease and poorer outcomes [22].

 Despite these numerous observations, the ESR level is not routinely incorporated into current prognostic models for RCC, likely due to the nonspecific nature of its elevation [24–26], and the relationship between ESR and survival in localized RCC following potentially curative nephrectomy has not been fully elucidated. We hypothesized preoperative ESR values were an independent prognostic indicator of overall survival in localized renal cell carcinoma following radical nephrectomy and could have potential benefit with respect to overall clinical management as well as preoperative patient counseling, especially constructing specific risk categories on the basis of ESR levels. 

## 2. Methods

 One hundred sixty-seven patients who underwent potentially curative radical nephrectomy (all macroscopic tumor was removed with negative surgical margins) for clear cell RCC had ESR measured preoperatively between November 2006 and February 2010. There were a total of 192 patients with RCC during this time period, 15% of which did not have a preoperative ESR value measured, mostly due to error in paperwork. Follow-up data was available through August 25, 2010. Patients underwent standard followup for post-nephrectomy RCC patients, including imaging studies every 3 months for 1 year, then spaced to every 6 months until 5 years postoperatively, then every year thereafter. Routine laboratory studies including CRP, serum creatinine, and ESR were checked every 3 months and physical exams were performed at office visits. Perioperative deaths (within 30 days of surgery) were excluded from analysis. Inclusion criteria consisted of clear cell histology, and exclusion criteria consisted of nodal or metastatic disease, or age less than 18 years. All patients underwent a cross-sectional imaging study (MRI or IV-contrasted CT) of the chest, abdomen, and pelvis before surgery. No patients received systemic therapy following nephrectomy. The Emory University Institutional Review Board approved this clinical database project.

Patients were staged pathologically according to the AJCC TNM renal tumor classification [27], and tumors were graded based on Fuhrman criteria [28]. Staging was initially based on six stages (T1a, T1b, T2, T3a, T3b, and T3c). However, one-way analysis of variance revealed no significant difference in outcomes between T1a and T1b and between T3a, T3b, and T3c. Additionally, there was no significant difference between T3 and T4 disease. Therefore, patients were divided into three groups based on T-stages: T1, T2, and T3-4.

Prior to surgery, clinical stage, routine laboratory measurements and ESR levels were assessed. The inter- and intra-assay variability for all laboratory values were <10%. Postoperatively, we assessed overall survival via Social Security Death Index. 

Frequency and descriptive analyses were conducted to characterize the patient population. Kaplan-Meier analysis was conducted to assess the univariate impact of these ESR-based risk groups on overall survival. Finally, univariate and multivariate Cox regression analysis was conducted to assess the potential of these groups to predict overall survival, adjusting for other patient and tumor characteristics. Statistical significance in this study was set at *P* < 0.05. All analyses were performed using SPSS version 16.0. 

## 3. Results

This study cohort consisted of 167 patients who underwent potentially curative nephrectomy for localized clear cell RCC. Receiver Operating Characteristics (ROC) curves were constructed and used to determine the Area Under the Curve (AUC) and relative sensitivity and specificity of preoperative ESR in predicting overall survival. From this curve, cut-offs for low risk (0.0–20.0 mm/hr), intermediate risk (20.1–50.0 mm/hr), and high risk (>50.0 mm/hr) groups were created. Of the total cohort, 101 patients (55.2%) were in the low risk group, while 40 patients (27.0%) and 26 patients (17.8%) were in the intermediate risk and high risk groups, respectively. The majority of patients in all risk categories were Caucasian males, with mean (SD) ages of 56.5 (±12.7) years, 64.4 (±14.2) years, and 64.2 (±11.7) years for the low, intermediate, and high risk categories, respectively (Table 1). The majority of patients in the low and intermediate risk groups had T1 disease, while higher T-stages predominated in the high risk group, with nearly half of these patients having either T3 or T4 RCC. Likewise, higher nuclear grades prevailed in the high risk group, with almost 70% of high risk patients having either Fuhrman nuclear grade III or IV. The mean (SD) ESR values were 10.1 (5.0) mm/hr, 31.5 (7.3) mm/hr, and 82.5 (24.9) mm/hr for the low, intermediate, and high risk groups, respectively (Table 1). 

Median (95% CI) survival for the ESR-based risk groups was 44.1 (42.6–45.5) months, 35.5 (32.3–38.8) months, and 32.1 (25.5–38.6) months for the low, intermediate, and high risk groups, respectively. Univariate and multivariate Cox regression analysis of overall survival showed a 3.2-fold (HR: 3.265, 95% CI: 0.993-10.733) increased risk of overall mortality for the intermediate risk group and a 8.4-fold (HR: 8.409, 95% CI: 2.740-25.805) increased risk for the high risk group. After controlling for patient age, race, gender, Charlson Comorbidity Index, T-Stage, Fuhrman Nuclear grade, and tumor size, intermediate risk and high risk groups experienced a 4.5-fold (HR: 4.509, 95% CI: 0.735–27.649, *P* = 0.033) and 18.5-fold (HR: 18.531, 95% CI: 2.117–162.228, *P* < 0.001) increased risk of overall mortality, respectively (Table 2).

**Figure 1:**
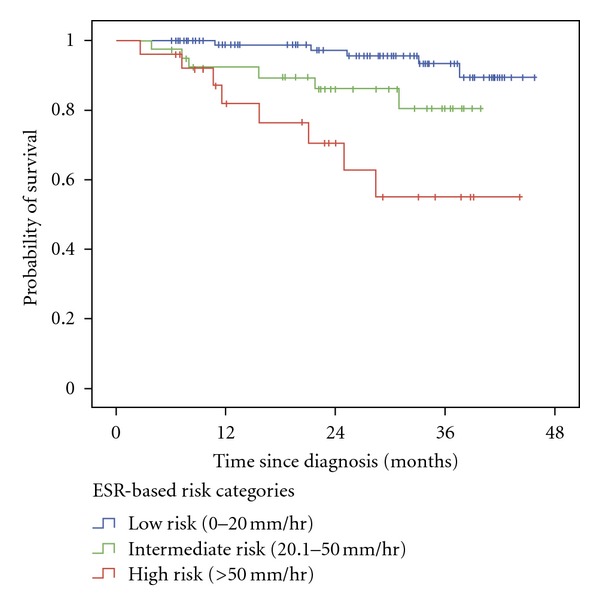
Kaplan-Meier survival analysis of probability of survival versus time since surgery (days) by preoperative ESR Risk Category among patients diagnosed with localized clear cell RCC undergoing potentially curative nephrectomy. Patients categorized into Low Risk (≤20.0 mm/hr), Intermediate Risk (20.1–50.0 mm/hr), and High Risk (>50.0 mm/hr) based on preoperative ESR levels. Log-rank: Low Risk versus High Risk (*P* < 0.001), Low Risk versus Intermediate Risk (*P* = 0.033), Intermediate Risk versus High Risk (*P* = 0.066).

 Kaplan-Meier survival analysis of probability of survival versus time since surgery stratified by preoperative ESR risk category into low, intermediate, and high risk groups showed a statistically significant difference in survival when comparing the low risk group to both the high risk group (*P* < 0.001) as well as comparing the low risk group to the intermediate risk group (*P* = 0.033). No statistically significant difference in survival was observed between the intermediate and high risk groups (*P* = 0.066), although a trend was observed (Figure 1). 

## 4. Discussion

Renal cell carcinoma can be ranked among the great masqueraders of clinical medicine, and its diagnosis at a stage early enough for curative nephrectomy remains a significant challenge. Clinically localized tumors are often symptom-free, and by the time clinical symptoms become apparent, more advanced tumors have a complex clinical course with increased morbidity and mortality. As such, numerous studies over several decades have focused on the identification of other objective measures for both diagnostic as well as prognostic use in defining risk groups for preoperative patient counseling and postoperative surveillance strategies [7, 12, 14, 22, 23, 29, 30].

 With the rapid and evolving understanding of renal tumor biology, RCC staging systems have likewise evolved over time. The first formal staging system dates to 1958, later modified by Robson in 1969 [31, 32]. Subsequent refinements have led to the development of the often-cited TNM classification, which stratifies patients' primary tumor into one of four classifications (I–IV). Similarly, this classification has also undergone multiple refinements since its inception in 1974. Regardless, many elements of the TNM staging system are cause for debate. This has led to the development of numerous integrated staging systems such as the UCLA/UISS (UCLA Integrated staging system) as well as the SSIGN (Stage, Size, Grade, and Necrosis) scoring algorithm [33]. Of note, none of these staging systems incorporates any measured serum markers. 

 Several recent studies have focused on the prognostic value of preoperative ESR levels in RCC following potentially curative nephrectomy for clinically localized disease [22, 23], as this is a quick and inexpensive laboratory test costing less than thirty dollars at our institution. 

In a recent meta-analysis, Wu and colleagues found the systemic inflammatory response to be a predictor of poor overall survival in patients with renal cell carcinoma [34]. In a total of 47 studies included for meta-analysis, the combined hazard ratios (HRs) for survival of CRP, platelet count (PC), and ESR were 3.46, 3.22, and 3.85, respectively. All three inflammatory indicators also predicted relapse-free survival (HRs > 2.0). 

Another recent study specifically analyzing the role of preoperative ESR values found that both tumor stage and preoperative ESR levels were both significant independent prognostic indicators of progression-free survival as well as disease-specific survival [23]. When analysis was limited to pT1 tumors, only ESR was an independent prognostic factor for disease-specific survival. 

 Two recent studies from the Mayo Clinic have reported elevated preoperative ESR levels portended an increased risk of death from RCC [7, 22], however, neither of these studies stratified patients preoperatively into low, intermediate, or high risk based on their ESR level. 

 The incidence of elevated ESR in patients with RCC has been reported to range between 23% and 50% [22], and has been noted as an independent prognostic factor for disease-specific survival (DSS) as well as progression-free survival (PFS) following nephrectomy [23]. However, as noted previously, despite these observations ESR is not incorporated into current prognostic algorithms for RCC. This could be due to many factors, not the least of which is its nonspecific nature as well as the poorly understood mechanism by which it reaches elevated levels. Early studies on the prognostic significance of ESR were fraught with uncertainty owing mainly to a lack of histologic stratification and the relatively small number of patients in each series. These issues have been addressed in a more recent study of larger cohorts of patients grouped by histologic subtype from the Mayo Clinic [22]. This study evaluated the prognostic significance of preoperative ESR in 1075 patients who underwent nephrectomy for RCC over 30 years. These authors observed an association between elevated preoperative ESR (defined as >22 mm/hr in male patients and >29 mm/hr in female patients) and death from clear cell RCC, papillary RCC, and chromophobe RCC, with risk ratios of 3.6, 3.8, and 10.3, respectively. 

Urologists are long familiar with the use of serum markers to risk-stratify patients with cancer. For example, different levels of PSA prior to definitive local therapy can be helpful in predicting outcome, as well as the previously mentioned prognostic value of preoperative CRP in renal cell carcinoma. To our knowledge, the current study is the first to stratify patients based on preoperative ESR level into low, intermediate, and high risk categories based on overall survival following nephrectomy for localized RCC. In multivariate analysis, preoperative ESR levels were significantly associated with an increased risk of overall mortality, with the intermediate and high risk groups experiencing a 4.5-fold and 18.5-fold increased risk of overall mortality, respectively. These results support our hypothesis that preoperative ESR levels independently predict overall survival following nephrectomy for clinically localized RCC and reinforce other studies asserting its prognostic significance. 

 This distinction is of paramount importance in the preoperative counseling of patients, and in helping to identify those most suitable for intense monitoring for postoperative disease recurrence as well as potential consideration for inclusion into adjuvant therapy trials. Unfortunately, until an adjuvant therapy demonstrates efficacy in those patients most at risk for recurrence, there is little to offer other than aggressive surgical therapy. 

 This study is limited by both its relatively small cohort of patients as well as the limited followup period. This is especially reflected in the wide confidence intervals. Furthermore, we did not account for lifestyle and socioeconomic variables, including BMI, alcohol or tobacco use, and diet, which could be confounding variables. We must also discuss the likely selection bias inherent in performing this type of study at a large, tertiary-care facility, where a large referral base naturally results in a larger proportion of patients with aggressive disease characteristics as seen in Table 1. Without question, further investigation is needed to fully clarify the role of preoperative ESR levels in the prognostication of patients with clinically localized RCC, as well as to determine the prognostic utility of postoperative values. However, there is clearly an association of elevated preoperative levels of ESR with poor overall outcomes. Further studies would need to investigate this conclusion over a longer period of time and among different patient populations. Nonetheless, we feel these findings are significant and suggest an expanded role for this simple and inexpensive preoperative laboratory assessment. 

 In conclusion, the erythrocyte sedimentation rate is an easily obtainable, relatively inexpensive serum marker whose level independently segregates patients with clinically localized renal cancer into different risk groups with significant differences in overall survival. Inclusion into nomograms may prove beneficial if this data is confirmed in larger and more varied study populations.

## Figures and Tables

**Table 1:** Patient characteristics.

Variables	Low risk (≤20.0 mm/hr) (*n* = 101)	Intermediate risk (20.1–50.0 mm/hr) (*n* = 40)	High risk (≥50.1 mm/hr) (*n* = 26)	All patients (*n* = 167)
Age (y)				
Mean (SD)	56.5 (12.7)	64.4 (14.2)	64.2 (11.7)	59.4 (12.8)
Race (%white/%nonwhite)	84.3/15.7	79.4/20.6	60.0/40.0	72.1/27.9
Gender (%male)	70.3	52.5	50.0	64.8
Charlson Comorbidity Index				
Mean (SD)	2.9 (1.6)	3.0 (1.7)	4.0 (2.3)	3.1 (1.7)
				
T-Stage (%T1/%T2/%T3-4)	84.3/7.9/7.9	68.4/10.5/21.1	35.5/16.7/45.8	72.3/10.3/17.4
Fuhrman Nuclear Grade				
(%I-II/%III/%IV)	59.1/39.8/1.1	50.0/44.7/5.3	29.2/54.2/16.7	49.5/43.2/7.3
Tumor size (cm)				
Mean (SD)	4.4 (2.6)	4.9 (2.9)	7.0 (4.3)	5.0 (3.2)
ESR^† ^(mm/hr)				
Mean (SD)	10.1 (5.0)	31.5 (7.3)	82.5 (24.9)	28.5 (29.4)

^†^Erythrocyte Sedimentation Rate.

**Table 2:** Univariate and multivariate Cox regression analyses of predictors of overall survival (OS).

Variable	Crude HR	95% CI	Adjusted HR	95% CI
ESR^†^-Based Risk Categories				
Low risk	Reference		Reference	
Intermediate risk	3.265	0.993–10.733	4.509^∗^	0.735–27.649
High risk	8.409	2.740–25.805	18.531^∗∗^	2.117–162.228
Age	1.028	0.992–1.065	1.030	0.971–1.093
Gender				
Female	Reference		Reference	
Male	1.502	0.610–3.697	1.306	0.322–5.298
Race				
White	Reference		Reference	
Non-white	0.658	0.149–2.896	0.150	0.018–1.243
Stage				
1	Reference		Reference	
2	1.675	0.195–14.374	0.761	0.050–11.573
3-4	10.077	3.436–29.552	4.685	0.721–30.449
Charlson Comorbidity Index	1.274	1.098–1.480	0.754	0.506–1.123
Grade				
1-2	Reference		Reference	
3	3.266	0.883–12.076	1.373	0.222–8.493
4	32.595	7.684–138.264	21.902	1.937–247.590
Tumor size	1.176	1.072–1.290	1.001	0.820–1.221

^†^ Erythrocyte Sedimentation Rate.

^∗^ *P* = 0.033.

^∗∗^ *P* < 0.001.
